# The Association Between Hip-Shoulder Separation Angles and Technique Characteristics in World-Class High Jumpers

**DOI:** 10.3389/fspor.2022.873526

**Published:** 2022-05-25

**Authors:** Gareth Nicholson, Nils Jongerius, Catherine B. Tucker, Aaron Thomas, Stéphane Merlino, Athanassios Bissas

**Affiliations:** ^1^Carnegie School of Sport, Leeds Beckett University, Leeds, United Kingdom; ^2^European School of Physiotherapy, Amsterdam University of Applied Sciences, Amsterdam, Netherlands; ^3^International Relations & Development Department, World Athletics, Monte Carlo, Monaco; ^4^Athletics Biomechanics, Leeds, United Kingdom; ^5^School of Sport and Exercise, University of Gloucestershire, Gloucester, United Kingdom

**Keywords:** kinematics, trunk, videography, rotation, competition

## Abstract

Hip-shoulder separation (H-S_sep_) has been widely researched in many sporting activities (e.g., golf) to provide information on the contribution of torso rotation to performance and injury. Although it is necessary for high jumpers to generate significant long-axis rotation to successfully clear the bar, limited information exists on H-S_sep_ for high jump athletes. As such, this study aimed to (a) characterize the H-S_sep_ of world-class high jump athletes during competition, (b) determine if differences exist between male and female athletes and (c) to examine the relationship between H-S_sep_ and the biomechanical parameters used to describe high jump technique. Twenty-nine world-class high jumpers (17 males, 12 females) were video recorded (frame rate: 120–200 Hz) during the 2017 and 2018 World Athletics Championship finals. H-S_sep_ was quantified at touchdown (TD) and take-off (TO) following manual digitizing (SIMI motion) and a number of other common biomechanical parameters were computed. The observed levels of H-S_sep_ at TD (−46±12°) and TO (16 ±11°) were in line with those reported previously for other sports. The magnitude of H-S_sep_ varied between individuals and showed significant associations with other approach and take-off characteristics. Significant differences in H-S_sep_ were not evident between male and female athletes despite significant differences in other performance- and technique-related parameters. These findings highlight the divergent take-off characteristics of world-class performers and their reliance on hip-shoulder interactions when generating long axis rotation. Coaches should be mindful of the mechanical and physical consequences of H-S_sep_ when developing technical models, conditioning interventions and coaching strategies.

## Introduction

The main objective of the high jump event is for the jumper to raise their center of mass (CM) to a maximum height whilst crossing the bar. As the high jump event has been contested since the first modern Olympiad it is not surprising that a great number of techniques have been adopted in order to achieve this (Schiffer, 2009). Although almost all modern high jumpers utilize the Fosbury Flop technique (Dapena, 2002), the technique still allows a wide range of technical variations compared to other jumps. There are a wealth of biomechanical data available for the high jump which characterizes the approach run, take-off and flight phases (Antekolovic et al., 2006; Coh and Supej, 2008; Nicholson et al., 2018, 2019); this forms the basis of current technical models used by coaches to develop strategies to achieve maximum technical efficiency.

Several authors have stated that the take-off is the most important phase of the high jump (Dapena, 2006) with the peak height of the CM during flight heavily dependent on the height and vertical velocity of the CM at take-off. Although a great deal of attention has rightly been focused on the critical determinants of these take-off parameters (Alexander, 1990; Dapena et al., 1990; Grieg and Yeadon, 2000), it is clear that the characteristics of the approach run, take-off and flight must also permit a supine layout position at the peak of the jump to enable successful bar clearance. It is well-known that high jump athletes utilize rotation around the frontal and longitudinal axes in order to adopt successful bar clearance positions. Although it is acknowledged that the actions a jumper makes in the air can impact on the bar clearance position, long axis rotation is influenced by the position and swinging action of the lead leg and also the turning of the shoulders and arms during take-off (Dapena, 1997). Problems with torso-rotation strategies may make it more difficult to move from a forward facing take-off position to a supine bar clearance position, as characterized by a tilted position at the apex of the jump. The need for torso rotation begins during the pre-take-off phase at a time when a jumper is also working to optimize the vertical velocity of the CM at take-off. This adds to the complexity of the movement and raises the potential for rotation-generation strategies to also negatively influence the peak height of the CM during flight. Whilst the generation of long axis rotation (*via* torso rotation) likely necessitates a high level of trunk strength and flexibility as well as notable technical proficiency, it is the power, reactive strength and stiffness of the lower body that are more widely cited as important physical characteristics for high jump success (Ridzdorf, 2009; Boden et al., 2017).

The hip-shoulder separation (H-S_sep_) parameter, defined as the angular displacement between the hips and shoulders has been widely used (Campos et al., 2004; Myers et al., 2008; Fleisig et al., 2013; Beach et al., 2016) to quantify trunk rotation in a variety of sports (e.g., javelin, golf, baseball, cricket). Greater H-S_sep_ in the wind-up phase of many striking sports is believed to facilitate increased stored energy utilization around the scapulae (Young et al., 1998), resulting in optimized rotation of the upper trunk when combined with activation of the oblique muscles to decelerate pelvis rotation (Fleisig et al., 2013). Whilst greater H-S_sep_ has been positively associated with increased club head velocity and ball velocity (Myers et al., 2008), repetitive asymmetric trunk rotation may lead to injuries in the lumbar spine (Glazier, 2010; Hsu, 2011; Fleisig et al., 2013). Despite the fact that trunk rotation generated through hip-shoulder interaction plays at least some role in generating long axis rotation in the high jump (Dapena, 1997), biomechanical data relating to hip-shoulder interactions are scarcely reported. Dapena (1997) used video data from 10 elite high jumpers (5 male, 5 female) and through the use of computer simulation, reported differing rotation strategies (i.e., pre take-off vs. airborne actions) of male and female jumpers due to the fact the lower jump height of female jumpers allowed a much shorter time for rotation.

Whilst the differences in trunk contributions between male and female jumpers may have important implications for coaches, biomechanical analyses in competitive environments have since then focused on traditional parameters relating to the speed and shape of the approach, temporal and postural (lower-body) characteristics of the take-off and basic descriptors (e.g., peak CM height) of the flight phase (Antekolovic et al., 2006; Isolehto et al., 2007; Ae et al., 2008; Coh and Supej, 2008). As a result, very limited information exists on the strategies used by modern, world-class high jumpers to generate long axis rotation and optimal layout positions for bar clearance. Considering that modern, world-class high jumpers display great variation in the speed and shape of the approach run, the take-off time and distance and the bar clearance style (Nicholson et al., 2018, 2019), it would be useful to understand the constraints that these technique variants place on hip-shoulder interactions and trunk rotation before take-off. Such information would (a) improve the accuracy of our technical models as reference points for coaches, (b) inform the design of corrective strategies relating to bar clearance and (c) facilitate coaches in developing techniques that best suit the physical characteristics of each athlete.

At present there is limited information that describes hip-shoulder interactions before take-off in world-class high jump athletes which may play an important role in generating long axis rotation for effective bar clearance. Furthermore, the influence that different technical interpretations have on hip-shoulder interactions is also unclear despite the range of technical models that exist for the high jump event. Whilst there is limited research across all standards of high jump athletes, research into the highest level of performers can be used by coaches as models of excellence to inform their technical and conditioning strategies. Thus, the first aim of this study was to describe the H-S_sep_ of world-class male and female high jump athletes before take-off during the finals of the 2017 and 2018 World Athletics Championships. The second aim was to investigate the relationship between H-S_sep_ and biomechanical parameters used to describe the speed and shape of the approach, spatiotemporal and postural characteristics of take-off and the bar clearance style. A better understanding of trunk rotation and technical interpretations provides scientists, coaches and conditioners with the means to optimize technical solutions, physical preparation, and injury prevention.

## Methods

### Participants

Data were collected as part of the Birmingham 2018 IAAF World Indoor Championships (1st March) and London 2017 World Outdoor Championships (13th August) Biomechanics Projects. The use of the data for this study was approved by World Athletics, who own and control the data, and locally through institutional research ethics procedures. The study was approved by the Leeds Beckett University Ethics sub-committee (application reference: 61250). The best jumps from twenty-nine world-class high jumpers were analyzed (body mass: 70.89 ± 8.31 kg; height: 1.88 ± 0.08 m; personal best: 2.19 ± 0.18 m) which included seventeen male athletes (body mass: 76.44 ± 5.50 kg; height: 1.92 ± 0.04 m; personal best: 2.33 ± 0.04 m) and twelve female athletes (body mass: 63.50 ± 4.89 kg; height: 1.82 ± 0.07 m; personal best: 1.99 ± 0.03 m). These athletes were selected as the finalists for the men's and women's events for the Birmingham and London championships. Athletes who competed in both competitions were only analyzed once.

### Protocol

Five high-speed cameras were employed to record the action during the high jump finals at the 2017 outdoor [Sony RX10 M3 cameras operating at 120 Hz [shutter speed: 1/1600 s; resolution: 1920 x 1080 [progressive] pixels]] World Championships and four high-speed cameras [Sony PXW-FS5 cameras operating at 200 Hz [shutter speed: 1/1250 s; resolution: 1920 x 1080 [progressive] pixels]] were used to record the high jump action during the 2018 indoor World Championship. The cameras were positioned strategically in pairs with their optical axes positioned to enable a three-dimensional analysis of each athlete's jump. Full-body motion capture took place commencing three steps before take-off and ending when the athlete had landed. The same standardized calibration procedure was conducted before and after each final. A rigid cuboid calibration frame measuring 3.044 x 3.044 x 3.044 m and comprising 24 reference points was used. It was sequentially repositioned multiple times in discrete predefined areas to create an accurate defined volume covering the high jump run-up and take-off area.

### Data Processing

The video files were imported into SIMI Motion (SIMI Motion version 9.2.2, Simi Reality Motion Systems GmbH, Germany) and the highest successful attempt for each athlete was manually digitized by a single experienced operator to obtain kinematic data. An event synchronization technique (synchronization of four critical instants) was applied through SIMI Motion to synchronize the two-dimensional coordinates from each camera involved in the recording. The digitizing involved a continuous whole-body analysis throughout the approach, take-off and flight phases of each jump. A 17-point whole body model was digitized beginning three steps from the final take-off position and ending following complete bar clearance by each athlete. In accordance with de Leva (1996), the 17 digitized points were the center of the head, and bilaterally shoulder, elbow, wrist, metacarpo-phalangeal, hip, knee, ankle and metatarso-phalangeal (MTP) joint center. Each file was first digitized frame by frame and upon completion adjustments were made as necessary using the points over frame method (Bahamonde and Stevens, 2006). The reliability of the digitizing process showed minimal total errors (ICC > 0.97) when it was repeated for specific variables for five randomly selected athletes with an intervening period of 48 h. The Direct Linear Transformation (DLT) algorithm (Abdel-Aziz and Karara, 1971) was used to reconstruct the real-world 3D coordinates from individual camera's x and y image coordinates. de Leva (1996) body segment parameter models were used to obtain data for the whole-body center of mass and for key body segments. A recursive second-order, low-pass Butterworth digital filter (zero phase-lag) was employed to filter the outcome variable with a 7–15 Hz cut-off frequency range determined through residual analysis (Winter, 2009).

Following data processing, shoulder-hip separation angles (H-S_sep_) were calculated using a custom-written Matlab script (version R2021b, MathWorks, Inc., Natick, MA). A line connecting the mid-point of left and right hip markers to the mid-point of left and right shoulder markers defined the transverse axis. Rotation around the axis was investigated by calculating H-S_sep_ as the angle created by a line connecting both shoulders relative to a line connecting both hips around this transverse axis. The calculation of H-S_sep_ took place from touchdown (TD) until toe-off (TO) for the final ground contact phase before take-off in each jump, this enabled the characterization of H-S_sep_ throughout the take-off phase of the event. In addition to the computation at discrete events at TD and TO, H-S_sep_ was normalized to 101 datapoints throughout the take-off phase for each jump to provide comparative data on H-S_sep_ modifications. In order to allow the association between H-S_sep_ and technique characteristics to be determined, a number of commonly measured kinematic variables were also computed to characterize the approach, take-off and flight phases of each athlete's jump (Table 1). Athlete's heights were obtained (Matthews, 2017) and used to scale a number of linear displacement (horizontal and vertical) variables to account for differences in stature.

**Table 1:** Definitions for all variables analyzed.

**Variable**	**Definition**
Mark	The mark attained during competition.
Hip-shoulder separation angle	The angle between a vector joining the right and left hips and a vector connecting the right and left shoulders.
Hip orientation	The angle of a vector joining the right and left hips relative to the upright. 90°= perpendicular with the upright
Shoulder orientation	The angle of a vector joining the right and left shoulders relative to the upright. 90°= perpendicular with the upright
CM height	The vertical height of the CM at TD and TO during the final foot contact.
Take-off distance	The foot-tip distance (anteroposterior) from the bar at take-off.
Knee distance	The horizontal distance (resultant) between the left and right knee joints.
CM attack angle	The angle between the peak CM position (during bar clearance) and the CM position at final take-off.
Step-to-bar angle	The angle between respective foot contacts relative to the bar.
Flight distance	The horizontal distance (resultant) between the CM at TO (during the take-off phase) and the CM at peak height (during the flight phase).
Step length	The displacement between toe-off of consecutive foot contacts.
Contact time	The time spent in contact with the ground during foot contact.
Horizontal CM velocity	The horizontal velocity (resultant of anteroposterior and mediolateral components) of the CM.
Vertical CM velocity	The vertical velocity of the CM at different time instants.
ΔCM velocity	The percentage change in CM velocity between the TD and TO of the take-off phase.
Take-off angle	The angle of the CM relative to the horizontal at the instant of take-off.
Knee angle	The angle of the thigh relative to the shank (180°= full extension).
Ankle angle	The angle of the shank relative to the foot (180°= full plantar flexion).
Peak CM height	The maximum vertical height of the CM during bar clearance.
Time to peak height	The time period between TO and peak CM height (during bar clearance).

*CM, center of mass; TD, touchdown; TO, take-off*.

### Statistics

Results are reported as means ± standard deviation (SD). All statistical analyses were carried out using SPSS Statistics 26 (IBM SPSS, Inc., Chicago, IL) and distribution parameters were used to check the appropriateness of parametric tests. Independent samples *t*-tests were used to compare differences between male and female athletes for all dependent variables, significance was set at *p* < 0.05 (Field, 2009). Cohen's *d* (Cohen, 1988) was used as an effect size to determine the magnitude of differences between groups with interpretation thresholds of 0.2 (small), 0.5 (medium), 0.8 (large), 1.2 (very large), and 2.0 (huge). Additionally, Pearson's correlations (two-tailed) were used to establish associations between H-S_sep_ and technique characteristics with magnitudes interpreted according to the guidelines of Hopkins et al. (2009).

## Results

Table 2 shows the mean H-S_sep_ angles at touchdown and take-off for the full cohort. Table 2 shows that men and women displayed no significant differences in H-S_sep_ angles at TD (men: −47.35 ± 12.48, women: −43.58 ± 10.04) and TO (men: 15.88 ± 11.00, women: 16.00 ± 13.91) and no significant differences in H-S_sep_ range of motion (men: 63.24 ± 12.94, women: 59.58 ± 15.12) between the two time points.

**Table 2:** Variables representing the orientation of the shoulders and hips at touchdown (TD) and take-off (TO) of the final ground contact phase for men, women and the combined sample.

**Variable**	**Combined**	**Men**	**Women**	**Effect Size (*d*)**
Shoulder-Hip Separation Angle TD (°)	−46 ± 12 (−66 to −22)	−47 ± 12 (−66 to −26)	−44 ± 10 (−56 to −22)	0.33
Shoulder-Hip Separation Angle TO (°)	16 ± 11 (41 to 2)	16 ± 9 (41 to 2)	16 ± 14 (41 to 2)	0.01
Shoulder-Hip Separation ROM (°)	62 ± 14 (84 to 30)	63 ± 13 (80 to 42)	60 ± 15 (84 to 30)	0.26

*Values are mean ± SD with upper and lower ranges shown in brackets. The range of motion (ROM) from touchdown to take-off is also shown for each group*.

Table 3 shows that the men achieve a significantly higher mark than women (2.25 ± 0.05 vs. 1.95 ± 0.04, *p* < 0.001). In terms of take-off characteristics, the men were characterized by a significantly greater CM height at TO (*p* < 0.01), had a greater take-off distance (*p* < 0.01) and final step length (*p* < 0.05) and had a greater knee distance at TD (*p* < 0.001) than their female counterparts. There were no differences in joint kinematics at TD or TO but the men did display a significantly higher vertical CM velocity at TO (*p* < 0.001), a larger take-off angle (*p* < 0.01) and higher horizontal CM velocity at TD (*p* < 0.001) and larger change in CM velocity during the take-off phase (*p* < 0.01). In terms of flight characteristics, the men displayed a higher peak CM height (*p* < 0.001), a longer time to peak height (*p* < 0.001) and a greater flight distance than the female athletes (*p* < 0.001).

**Table 3:** Variables (mean ± SD) representing the kinematic and temporal characteristics of the take-off and flight phases of the jumps for the men, women and combined sample.

**Variable**	**Combined**	**Men**	**Women**	**Cohen's *d***
**General characteristics**				
Body mass (kg)	70.89 ± 8.31	76.44 ± 5.50^***^	63.50 ± 4.89	2.49
Stature (m)	1.88 ± 0.08	1.92 ± 0.04^***^	1.82 ± 0.07	1.93
Mark (m)	2.12 ± 0.16	2.25 ± 0.05^***^	1.95 ± 0.04	6.23
**Take-off characteristics**				
CM height at TD (m)	0.89 ± 0.05	0.90 ± 0.05	0.88 ± 0.05	0.41
CM height at TO (m)	1.30 ± 0.07	1.33 ± 0.06^**^	1.25 ± 0.06	1.28
Take-off distance (m)	1.07 ± 0.27	1.19 ± 0.26^**^	0.90 ± 0.18	1.32
Knee distance at TD (m)	0.47 ± 0.08	0.51 ± 0.06^***^	0.41 ± 0.06	1.75
CM attack angle at TO (°)	30.5 ± 12.1	28.9 ± 11.4	32.6 ± 13.1	0.30
Step-to-bar angle (°)	31.0± 6.7	30.6 ± 6.8	31.4 ± 6.9	0.12
Penultimate step length (m)	2.07 ± 0.18	2.11 ± 0.19	2.02 ± 0.15	0.54
Final step length (m)	1.94 ± 0.22	2.01 ± 0.13^*^	1.83 ± 0.28	0.88
Final contact time (s)	0.16 ± 0.03	0.16 ± 0.01	0.15 ± 0.01	0.14
Horizontal CM velocity at TD (m/s)	7.23 ± 0.54	7.61 ± 0.36^***^	6.70 ± 0.18	3.37
Vertical CM velocity at TO (m/s)	4.36 ± 0.39	4.65 ± 0.20^***^	3.96 ± 0.17	3.73
Horizontal CM velocity at TO (m/s)	4.14 ± 0.37	4.19 ± 0.37	4.07 ± 0.37	0.32
ΔCM velocity (%)	−45.58 ± 5.29	−44.92 ± 4.13^**^	39.26 ± 5.10	1.23
Take-off angle (°)	46.5 ± 3.3	48.1 ± 2.6^**^	44.3 ± 2.9	1.39
Knee angle (trail leg) TD (°)	96.70 ± 12.46	98.89 ± 12.56	93.59 ± 12.17	0.43
Minimum knee angle (trial leg) (°)	37.40 ± 17.15	35.77 ± 16.97	39.70 ± 17.90	0.23
Knee angle (trial leg) TO (°)	77.26 ± 16.67	76.50 ± 16.03	78.35 ± 18.20	0.11
Knee angle (take-off leg) TD (°)	164.1 ± 5.38	164.6 ± 4.4	163.4 ± 6.7	0.22
Minimum knee angle (take-off leg) (°)	138.1 ± 6.5	137.8 ± 6.2	138.7 ± 7.2	0.13
Knee angle (take-off leg) TO (°)	169.5 ± 5.3	169.4 ± 6.2	169.5 ± 4.2	0.03
Ankle angle (take-off leg) TD (°)	123.4 ± 7.0	121.2 ± 5.4	126.6 ± 8.1	0.81
Minimum ankle angle (take-off leg) (°)	105.9 ± 6.8	105.0 ± 7.2	107.3 ± 6.4	0.35
Ankle angle (take-off leg) TO (°)	138.0 ± 6.8	137.9 ± 8.4	138.3 ± 4.0	0.07
**Flight characteristics**				
Peak CM height (m)	2.19 ± 0.16	2.32 ± 0.06^***^	2.01 ± 0.04	5.98
Time to peak height (s)	0.43 ± 0.04	0.45 ± 0.03^***^	0.40 ± 0.03	2.04
Flight distance (m)	1.74 ± 0.20	1.84 ± 0.18^***^	1.60 ± 0.10	1.70

*TD, Touchdown; TO, take-off*.

* *Significant difference between men and women (p < 0.05)*,

** *significant difference between men and women (p < 0.01)*,

*** *significant differences between men and women (P < 0.001)*.

In terms of the correlational analysis, no significant correlations were observed between any of the H-S_sep_ parameters and the mark attained by the athletes. However, Table 4 shows a number of significant correlations between the variables representing hip-shoulder orientation and the temporal and kinematic characteristics of the take-off and flight phases of the jumps. In particular, significant correlations were observed between H-S_sep_ at TD and the H-S_sep_ ROM (*r* = 0.63, *p* < 0.01), knee distance at TD (*r* = −0.47, *p* < 0.01), vertical CM velocity at TO (*r* = −0.40, *p* < 0.05) and the knee angle at TD (*r* = −0.44, *p* < 0.05). For H-S_sep_ at TO, a significant correlation was observed with H-S_sep_ ROM (*r* = −0.59, *p* < 0.01). Finally, H-S_sep_ ROM displayed significant correlations with H-S_sep_ at TD (*r* = 0.63, *p* < 0.01), H-S_sep_ at TO (*r* = −0.59, *p* < 0.01) and the knee distance at TD (*r* = −0.47, *p* < 0.47).

**Table 4:** Correlation coefficients (full cohort) between technique-related variables and hip-shoulder positioning parameters at touchdown and toe-off.

**Variable**	**H-S_sep_ TD (°)**	**H-S_sep_ TO (°)**	**H-S_sep_ ROM (°)**
H-S_sep_ TD (°)	1.00	0.25	0.63^**^
H-S_sep_ TO (°)	0.25	1.00	−0.59^**^
H-S_sep_ ROM (°)	0.63^**^	−0.59^**^	1.00
Mark (m)	−0.32	−0.15	0.15
CM height at TD (m)	0.17	−0.01	−0.15
CM height at TO (m)	0.04	−0.04	−0.06
Take-off distance (m)	−0.08	0.08	−0.13
Knee distance at TD (m)	−0.47^**^	0.10	−0.47^**^
CM attack angle at TO (°)	−0.14	−0.08	−0.05
Step-to-bar angle (°)	−0.23	−0.31	0.06
Flight distance (m)	−0.23	−0.03	−0.17
Penultimate step length (m)	0.14	−0.04	0.14
Final step length (m)	−0.12	0.16	−0.23
Final contact time (s)	−0.08	−0.08	0.00
Horizontal CM velocity at TD (m/s)	−0.20	0.01	−0.17
Vertical CM velocity at TO (m/s)	−0.40^*^	−0.05	−0.29
Horizontal CM velocity at TO (m/s)	−0.09	0.09	−0.15
ΔCM velocity (%)	0.08	0.10	−0.02
Take-off angle (°)	−0.25	−0.12	−0.11
Knee angle (trail leg) TD (°)	−0.14	0.20	−0.28
Minimum knee angle (trial leg) (°)	0.10	0.33	−0.18
Knee angle (trial leg) TO (°)	0.07	0.19	0.09
Knee angle (take-off leg) TD (°)	−0.44^*^	−0.06	−0.32
Minimum knee angle (take-off leg) (°)	−0.09	0.01	−0.08
Knee angle (take-off leg) TO (°)	0.26	0.22	0.04
Ankle angle (take-off leg) TD (°)	−0.12	−0.14	0.01
Minimum ankle angle (take-off leg) (°)	−0.01	0.04	−0.04
Ankle angle (take-off leg) TO (°)	−0.24	−0.01	−0.19
Peak CM height (m)	−0.28	−0.11	−0.14
Time to peak height (s)	−0.39	−0.03	−0.31

* *Association significant at the p < 0.05 level*,

** *association significant at the p < 0.01 level*.

## Discussion

The purpose of this study was to (a) characterize the H-S_sep_ of world-class high jump athletes during competition, (b) determine if differences exist between male and female athletes and (c) to examine the relationship between H-S_sep_ and the biomechanical parameters used to describe high jump technique. The data demonstrate that modern athletes make use of hip-shoulder interactions throughout the take-off phase of the high jump with the magnitude of this interaction showing variation between individuals and significant associations with other biomechanical parameters used to describe the approach and take-off characteristics. Significant differences in H-S_sep_ were not evident between male and female athletes despite significant differences in other performance- and technique-related parameters.

The H-S_sep_ parameter has been widely used to quantify trunk rotation in a variety of sports (Campos et al., 2004; Myers et al., 2008; Fleisig et al., 2013; Beach et al., 2016). A previous investigation in recreational golfers (Myers et al., 2008) reported torso-pelvic separation values at the top of the backswing (−44*to* − 49°) that are comparable to the present investigation. Similarly, Boden et al. (2017) reported maximal axial rotation in professional baseball pitchers (55 ±6°) before ball release. H-S_sep_ in these striking sports is believed to facilitate increased stored energy utilization around the scapulae (Young et al., 1998) and has been positively associated with increased club head velocity and ball velocity (Myers et al., 2008). As expected, the magnitude of H-S_sep_ at touchdown (−46±12°) and take-off (16 ±11°) in the high jump was comparable with the values reported for the other sports. In the high jump, it has previously been suggested (Dapena, 1997) that the turning of the shoulders and arms and the swinging action of the lead leg influences an athlete's ability to generate long axis rotation although there is a scarcity of data quantifying hip-shoulder interactions in high jumpers, particularly during competitive situations. The present study provides support for this theory with H-S_sep_ likely being critical within the Fosbury Flop technique to quickly move from a curved approach run to a bar clearance position in which the body is horizontal and perpendicular with the bar. Repeated hip-shoulder interactions of this kind likely require high levels of trunk strength, flexibility and technical proficiency and should be considered by coaches working with high jump athletes to optimize performance and reduce injury.

The magnitude of H-S_sep_ necessary for optimal performance is likely dependent on the technical approach adopted by individual athletes. Indeed, a high degree of individual variability was displayed in the H-S_sep_ values at touchdown (from −66 *to* − 22°) and take-off (from 41 to 2°) and also in the H-S_sep_ range of motion throughout the take-off phase (from 84 to 30°). This no doubt reflects the great variation that modern, world-class high jumpers are known to display in the speed and shape of the approach run, the take-off time and distance and the bar clearance style (Nicholson et al., 2018, 2019). The variation in individual technical models likely necessitates specific H-S_sep_ requirements to facilitate effective bar clearance positioning however, no significant associations were observed between H-S_sep_ parameters, and any variables linked to the bar clearance phase, take-off distance or the shape or velocity of the approach.

The significant associations observed between H-S_sep_ at touchdown and take-off and the H-S_sep_ range of motion are not surprising given the interdependency of these metrics. The significant association between H-S_sep_ at touchdown and the vertical CM velocity at take-off (*r* = −0.40) is more interesting and would appear to highlight that greater H-S_sep_ at touchdown results in lower vertical velocities at take-off which play a key role in maximizing vertical CM displacement during flight (Dapena, 2006). The need for torso rotation begins pre take-off at a time when a jumper is also working to optimize the vertical velocity of the CM in preparation for take-off. It may be that the complexity of the movement during take-off causes a trade-off between the optimization of vertical propulsion and the generation of long-axis rotation however, such an interpretation should be treated with caution given the lack of association between H-S_sep_ parameters and the peak CM height during flight. In terms of the influence of other body positioning variables on H-S_sep_, only a handful of significant associations were observed. More specifically, the significant associations between H-S_sep_ at touchdown and the knee distance at touchdown (*r* = −0.47) and knee angle (take-off leg) at touchdown (*r* = −0.44) indicate that H-S_sep_ (TD) is greater when the distance between the two knees is smaller and the take-off leg is in a more flexed position. Together these correlations provide an important insight into the influence that H-S_sep_ has on lower-body positioning at the commencement of the take-off phase. It would be interesting to investigate the association between H-S_sep_ and other biomechanical variables further throughout transition phase of the approach to better understand the factors which permit hip-shoulder interaction. Nevertheless, coaches should be mindful of the associations between these biomechanical parameters when designing and implementing technical models and corrective strategies.

As expected, male athletes achieved a significantly greater bar clearance height than their female counterparts (2.25 vs. 2.12 m) despite their greater body mass, which likely resulted from their greater stature, physical characteristics and the subsequent technical models aligned to these factors. In terms of the latter, the greater bar clearance heights of the male athletes were characterized by significantly greater vertical CM velocities at take-off and significantly greater peak CM heights during the airborne phase. These performance characteristics were underpinned by faster horizontal CM velocities at touchdown, larger reductions in horizontal velocity during take-off and higher take-off angles which manifested despite greater take-off distances. Although there were no differences in the shape of the approach or final ground contact times, male athletes demonstrated a larger final step length and greater CM height at take-off which likely resulted from their greater stature. Despite the differences between males and females in a number of performance- and biomechanical-related parameters, there were no significant differences between males and females for any of the H-S_sep_ parameters analyzed. A previous investigation by Dapena (1997) which utilized a smaller sample size (5 males, 5 females) reported differing rotation strategies between male and female jumpers and linked these to the lower bar clearance heights of female athletes resulting in shorter time for rotation. Despite the significantly higher bar clearance heights of males along with longer times to peak CM height and greater (horizontal) flight distances, the present investigation suggests that trunk rotation plays a similar role in generating long axis rotation in male and female athletes. This finding may reflect the professionalization of (female) sport over the past 30 years which is likely to have reduced gender differences in technical proficiency. That being said, technical and performance differences do exist between males and females. Given the absence of pre-take-off differences in the strategies used to generate long axis rotation, further work is needed to better understand the approaches of male and female athletes. In particular, future data collection in competitive situations which focuses on the airborne phase of jumps would complement this investigation and provide a better understanding of the contribution of airborne actions to the generation of long axis rotation in male and female athletes.

The main strength of this study is that the data are of world-class high jump athletes (McKay et al., 2021) competing in World Championship finals, and therefore the research has high ecological validity, and the results can be used by coaches as a model of excellence. One possible limitation of the participant characteristics and the focus on only successful jumps is the limited sample size and the homogenous nature of the sample which may have reduced the number and/or magnitude of the relationships observed in the data. Future data collection which involves lower standards of athlete (i.e., trained, highly trained, elite) and unsuccessful trials may help to understand the key factors in high jump success further. Future biomechanical studies at world-class competitions that focus on the airborne phase of jumping and more steps in the approach would also complement these findings and provide more information to coaches on the pre-take-off and airborne contributions to long axis rotation generation in world-class high jump athletes. In addition, a future focus on intra-individual variation and the variation across performance levels will strengthen our understanding of the key factors governing success in the high jump event.

## Conclusions

World-class high jump athletes make use of hip-shoulder interactions during the take-off phase of jumps in order to generate long axis rotation within the Fosbury Flop technique. The between-individual variation in H-S_sep_ was expected and no doubt reflects the variety of technical variations available for the event. Given the associations between H-S_sep_ and other whole-body (e.g., CM vertical velocity) and joint-specific (e.g., knee flexion) parameters, coaches should be aware that torso rotation is a common strategy utilized by world-class high jumpers to prepare for bar clearance however, there are potential trade-offs in modifying torso rotation which may have specific implications in other areas of the movement. Since there are performance- and technique-related differences between males and females, but no differences in H-S_sep_ parameters, coaches should be mindful of other (e.g., airborne) strategies used to generate the long axis rotation when working with female athletes. Above all, these findings should be considered in the conditioning, injury prevention and technical strategies adopted when working with world-class high jump athletes.

## Data Availability Statement

The datasets presented in this article are not readily available because data part of externally funded project. Requests to access the datasets should be directed to GN: g.nicholson@leedsbeckett.ac.uk.

## Ethics Statement

The studies involving human participants were reviewed and approved by Leeds Beckett University Ethics Sub-Committee. The patients/participants provided their written informed consent to participate in this study.

## Author Contributions

GN, CT, NJ, AT, and AB performed data collection and processed the data. All authors conceptualized and designed the study, wrote the manuscript, interpreted the results of the research, edited, critically revised, and approved the final version for submission.

## Funding

The data collection and initial data analysis were supported by funding provided by the IAAF/World Athletics as part of a wider development/education project; however, the nature of the data is purely descriptive and not associated with any governing body, commercial sector, or product. The results of the present study do not constitute endorsement by World Athletics.

## Conflict of Interest

The authors declare that the research was conducted in the absence of any commercial or financial relationships that could be construed as a potential conflict of interest.

## Publisher's Note

All claims expressed in this article are solely those of the authors and do not necessarily represent those of their affiliated organizations, or those of the publisher, the editors and the reviewers. Any product that may be evaluated in this article, or claim that may be made by its manufacturer, is not guaranteed or endorsed by the publisher.
